# Exploring the Association of Surface Plasmon Resonance with Recombinant MHC:Ig Hybrid Protein as a Tool for Detecting T Lymphocytes in Mice Infected with* Leishmania (Leishmania) amazonensis*

**DOI:** 10.1155/2017/9089748

**Published:** 2017-03-08

**Authors:** Lenilton Silva da Silveira-Júnior, Franklin Souza-Silva, Bernardo Acácio Santini Pereira, Léa Cysne-Finkelstein, Geraldo Barroso Cavalcanti Júnior, Carlos Roberto Alves

**Affiliations:** ^1^Laboratório de Biologia Molecular e Doenças Endêmicas, IOC, Fiocruz, Av. Brasil 4365, 21040-900 Rio de Janeiro, RJ, Brazil; ^2^Laboratório de Imunologia Clínica, Departamento de Análises Clınicas e Toxicológicas, Faculdade de Farmácia, Centro de Ciências da Saúde, Universidade Federal do Rio Grande do Norte, Avenida Gustavo Cordeiro de Farias S/N, 59010-180 Natal, RN, Brazil; ^3^Laboratório de Imunoparasitologia, IOC, Fiocruz, Av. Brasil 4365, 21040-900 Rio de Janeiro, RJ, Brazil

## Abstract

A surface plasmon resonance- (SPR-) based recognition method applying H-2 L^d^:Ig/peptides complexes for ex vivo monitoring cellular immune responses during murine infection with* Leishmania (Leishmania) amazonensis* is described. Lymphocytes from lesion-draining popliteal lymph nodes were captured on a carboxylated sensor chip surface previously functionalized with H-2 L^d^:Ig (DimerX) protein bound to synthetic peptides derived from the COOH-terminal region of cysteine proteinase B of* L. (L.) amazonensis.* In computational analysis, these peptides presented values of kinetic constants favorable to form complexes with H-2 L^d^ at neutral pH, with a Gibbs free energy Δ*G*° < 0. The assayed DimerX:peptide complexes presented the property of attaching to distinct T lymphocytes subsets, obtained from experimentally infected BALB/c mice, in each week of infection, thus indicating a temporal variation in specific T lymphocytes populations, each directed to a different COOH-terminal region-derived peptide. The experimental design proposed herein is an innovative approach for cellular immunology studies of a neglected disease, providing a useful tool for the analysis of specific T lymphocytes subsets.

## 1. Introduction

According to the World Health Organization, leishmaniasis is one of the most neglected diseases in the world with extremely limited investments in the development of diagnosis, treatments, or control management (http://www.who.int/neglected_diseases/diseases/en). The disease is endemic in tropical and subtropical regions, covering more than 90 countries and territories, affecting mainly the population of developing countries, where more than 350 million people are at risk. It is estimated that 12 million cases exist worldwide, with 2 million new cases occurring each year: 0.5 million of visceral leishmaniasis (VL) and 1.5 million of cutaneous leishmaniasis (CL) [1, 2].

The term leishmaniasis comprises a set of clinical manifestations resulting from infection by the obligate intracellular protozoan parasites from the genus* Leishmania*. One species belonging to this genus is the* Leishmania (Leishmania) amazonensis*, which is an important etiologic agent of cutaneous leishmaniasis in humans, perhaps most importantly, with varied clinical manifestations in their infections [3, 4].

*Leishmania* parasites present virulence factors, which confers them the ability to invade and survive within the host, thus possibly causing diseases. Among these factors, we can highlight enzymes, like cysteine-proteinases (CPs), as playing relevant roles in the infection process. One specific CP, which has been reported as a pivotal virulence factor for species of the* L. (L.) mexicana* complex, is the cysteine proteinase B (CPB) that presents a characteristic extension of about 100 amino acids at its COOH-terminal region [4]. It has been proposed that, during the intracellular life stage of the parasite, fragments of COOH-terminal region of CPB* L. (L.) amazonensis* (cyspep) may interact with some factors of the immune system of the vertebrate host, including major histocompatibility complex (MHC) proteins [5–7].

In the present manuscript, we propose a new tool for monitoring the T lymphocytes-linked immune response during the evolution of* L. (L.) amazonensis* infection in mice. Our intent is to construct an adaptable methodology to analyze the molecular/cellular network of events related to antigen presentation in a murine infection.

## 2. Materials and Methods

### 2.1. Chemicals and Culture Media

Dimethylsulfoxide (DMSO), bovine sera albumin (BSA), *β*-mercaptoethanol (*β*-ME), hydroxyethyl piperazineethanesulfonic acid (HEPES), ethylenediaminetetraacetic acid (EDTA), RPMI medium, and Schneider's medium were purchased from Sigma-Aldrich Chemical Co. (St Louis, MO, USA). Fetal calf serum (FCS) was purchased from Gibco, Invitrogen (Brazil). Brain heart infusion (BHI) medium was purchased from Difco (Detroit, MI, USA). The synthetic peptides were produced using N-protected amino fluorenylmethoxy-carbonyl- (Fmoc-) based methodology, purchased from the GenScript Company. Recombinant Soluble Dimeric Mouse H-2L^d^:Ig (DimerX) protein and monoclonal antibodies conjugates [phycoerythrin-Cy-7 (PE-Cy7) Hamster anti-mouse CD3e, phycoerythrin (PE) Rat anti-mouse CD4, and fluorescein (FITC) Rat] were purchased from BD Biosciences (Becton, Dickinson and Company, Franklin Lakes, NJ, USA). Coupling agents [1-ethyl-3-(3-dimethylaminopropyl)carbodiimide (EDC) and N-hydroxysuccinimide (NHS)] and ethanolamide were purchased from Merck (Darmstadt, Germany). All other reagents were of analytical grade or superior.

### 2.2. Cultivation of Parasites

*L. (L.) amazonensis *(MHOM/BR/77/LTB0016) promastigotes (obtained from Coleção de* Leishmania *do Instituto Oswaldo Cruz (CLIOC), IOC, Fiocruz) were grown in Schneider's medium supplemented with 10% FCS for 4 days at 28°C. For the experimental infections, the parasites were washed 3 times with 50 mM phosphate buffered saline (PBS) pH 7.2, centrifuged at 2,000 ×g for 10 min at 4°C, counted using a Neubauer chamber, and resuspended in PBS at a concentration of 2.0 × 10^7^ cells/mL.

### 2.3. Mice and Experimental Infection

BALB/c 5–7-week-old female mice (H-2 haplotype^d^) were obtained from the animal care facility of Fiocruz (Centro de Criação de Animais de Laboratório (CECAL), Fiocruz). For the experimental infections, each animal was inoculated subcutaneously with 1.0 × 10^5^ stationary-phase promastigotes in PBS (50 *μ*L) in the left hind paw. For in vitro and ex vivo assays, cells were isolated from the lesion-draining popliteal lymph nodes of five mice infected with* L. (L.) amazonensis*. After isolating and counting the viable cells, these were adjusted and resuspended in RPMI medium supplemented with 2% FCS (5.0 × 10^5^ cells/mL). All procedures using animals were previously approved by the Animal Ethics Committee of Fiocruz (Comissão de Ética no Uso de Animais (CEUA), Fiocruz; L-0006/07).

### 2.4. Phenotypic Analysis of T Lymphocytes

The phenotype of the cells from* L. (L.) amazonensis*-infected and noninfected BALB/c mice was determined using 1.0 × 10^6^ lymph node cells. The cells were washed with PBS (500 ×g, 10 min, 4°C) and resuspended in PBS containing 0.05% sodium azide and 2% FBS. Specific monoclonal antibodies Pe-Cy^7^-labeled hamster anti-mouse CD3e IgG, PE-labeled rat anti-mouse CD4 IgG, and FITC-labeled rat anti-mouse CD8a IgG (BD Pharmingen, USA) were added to the cell suspensions, in a 1 : 100 dilution, and incubated in the dark for 60 min at 4°C. Finally, the cells were washed with PBS (500 ×g, 10 min, 4°C), resuspended in PBS containing 1% paraformaldehyde, and analyzed using a BD FACSAria™ flow cytometer (Becton, Dickinson and Company). For each sample, 2.0 × 10^4^ lymphocytes were recorded in list mode and registered on a logarithmic scale histogram. During data acquisition, the volume and inner complexity parameters of the events were controlled to match the typical features of murine lymphocytes. Data analysis was performed using the Summit 4.3 software (DAKO, Fort Collins, CO, USA).

### 2.5. Chemical Synthesis of Peptides

The cyspep-derived peptides were selected in silico using the SYFPEITHI software (http://www.syfpeithi.de), which uses a combinatory algorithm to predict the potential binding peptides to the H-2 motifs. The purity of the Fmoc-peptides was assessed using reversed-phase liquid chromatography, and the molecular weight was confirmed through mass spectrometry. For use in ex vivo assays, the peptides were completely dissolved at PBS containing 2.0 mg/mL DMSO and stored at −20°C until further use.

### 2.6. Surface Plasmon Resonance Assays

*Surface Functionalization of Sensor Chip (Steps  1 and 2)*. The functionalization process was performed in eight steps using running buffer (10 mM HEPES, 3 mM EDTA, 150 mM NaCl, and 0.005% Tween 20, pH 7.4) at a continuous flow rate (Fr): (i) injection of 10 *μ*L of 50 mM HCl (Fr = 10 *μ*L/min); (ii) injection of 100 *μ*L 10 mM CH_3_COOH (Fr = 50 *μ*L/min); (iii) injection of 150 *μ*L of coupling agents (1 : 1) 400 mM EDC : NHS (Fr = 15 *μ*L/min); (iv) injection of 100 *μ*L 10 mM CH3COOH (Fr = 50 *μ*L/min); (v) injection of 100 *μ*L (0.5 *μ*g) of DimerX H-2 L^d^:Ig (Fr = 10 *μ*L/min); (vi) injection of 100 *μ*L 10 mM CH3COOH (Fr = 50 *μ*L/min); (vii) injection of 150 *μ*L 100 mM ethanolamine (Fr = 20 *μ*L/min); and (viii) injection of 200 *μ*L 50 mM HCl (Fr = 50 *μ*L/min).

*Complex Formation of H-2 L* ^*d*^ *:Ig/Peptide (Step  3)*. Binding of peptides to protein DimerX was performed through the injection of 100 *μ*L of each synthetic peptide over the functionalized sensor chip (Fr = 10 *μ*L/min). The peptides (2 mg/mL) were diluted in filtrated PBS, which was also used as the running buffer. The concentration of peptides for binding to the DimerX was determined according to the manufacturer's instructions:

(1) $$\begin{array}{c} M_{p}=MH\cdot R\cdot \frac{D_{p}}{DH}, \end{array}$$

where *M*_*p*_ is micrograms of the peptide, MH is micrograms of H-2 in the reaction, *R* is molar excess (160 mol) between the peptide and protein H-2, *D*_*p*_ is molecular weight of the peptide, and DH is molecular weight of DimerX (250,000 Da).

*Interaction of the Cells with the Complex DimerX/Peptide (Step  4)*. The reflectance values were expressed as resonance units per second (RU/s). In these assays, the kinetics of association and dissociation between immobilized DimerX/peptide complexes and lymph node cells were determined. These assays were performed with 10^4^ cells suspended in PBS in a final volume of 100 *μ*L (Fr = 5 *μ*L/min).

*Regeneration of the Sensor Chips (Step  5). *The regeneration was performed by the injection of 250 *μ*L of 30 mM HCl (Fr = 50 *μ*L/min) into the system, followed by the injection of 200 *μ*L PBS (Fr = 50 *μ*L/min). After this procedure, only the DimerX molecules were left on the sensor chip surface; thus they were able to form new complexes with different peptides and interact again with cells.

### 2.7. Equations Used to Estimate Kinetic Data

*Affinity Constants and Gibbs Free Energy. *The affinity (KD) between DimerX and the peptides was obtained from the *k*_*a*_ and *k*_*d*_ data (see (2)), as determined by the analysis of the kinetics of interaction at 25°C in the SPR assays. The following calculation was used to obtain the KD for interactions that rapidly reach equilibrium:

(2) $$\begin{array}{c} KD=\frac{k_{d}}{k_{a}}. \end{array}$$

The formation of molecular complex between DimerX and peptides complex was also analyzed to calculate the Gibbs free energy of dissociation. This energy is related to the equilibrium constant (*K*_eq_) of the transition state between the product (DimerX/peptide) and its transient state. The values of *K*_eq_ were calculated using the *k*_*a*_ and *k*_*d*_ data obtained from the SPR assay at 25°C:

(3) $$\begin{array}{c} \Delta G^{{}^{\circ}}=-RT\ln\frac{1}{K_{eq}}. \end{array}$$

### 2.8. Statistical Analysis

To compare results, Student's *t*-test was applied, assuming equal variance between the samples. The assays were performed three times, and the data matrices were considered statistically distinct when *p* value was lower than 0.05.

## 3. Results and Discussion

The technology of surface biosensing is a new trend in the characterization of cell surface proteins [8], due to its flexible and powerful ability to detect biomolecular interactions [9]. This method has been successfully applied in the recognition of ligands in mammalian cells [10, 11] and in the detection of pathogenic microorganisms present in the environment or food [12]. The analysis by SPR is especially efficient to determine molecular binding events and has been used to assess antigen-antibody interactions and to detect cellular products, such as cytokines, in the supernatant of cultures [13–15].

Additionally, some processes of interaction between parasite and host that involve proteins have been studied with the use of this technology, as the interaction between heparin and circumsporozoite protein of malaria in the parasitic invasion of liver cells [16]. Recent studies by our research group, applying biosensing methods, proved that* L. (V.) braziliensis* promastigotes [17] and* Trypanosoma cruzi* epimastigotes and trypomastigotes [18] can bind to immobilized heparin, thereby, pointing to the presence of heparin receptors on the surface of these parasites.

The molecular immune network of mice is known to be subverted during infection by* Leishmania *parasites [5, 19]. To further understand the mechanisms through which the parasites can interfere with their host immune network, there is a need to develop methodologies able to simulate and to assess, at the molecular level, the interactions that occur during the infection process. Therefore, in the present work, we devised an experimental design that integrates biological and biophysical approaches to partially simulate key events of antigen presentation in experimental murine infection with* L. (L.) amazonensis*, without the need of using specific markers. With this strategy, it was possible to simulate some aspects of the antigen presentation via major histocompatibility complex (MHC) class I to T lymphocytes, once the used fusion protein has characteristics that allow for reproducing in vitro, with reasonable accuracy, the conditions in which epitopes/MHC/T cells receptor interactions occur in vivo [20].

**Figure 1:**
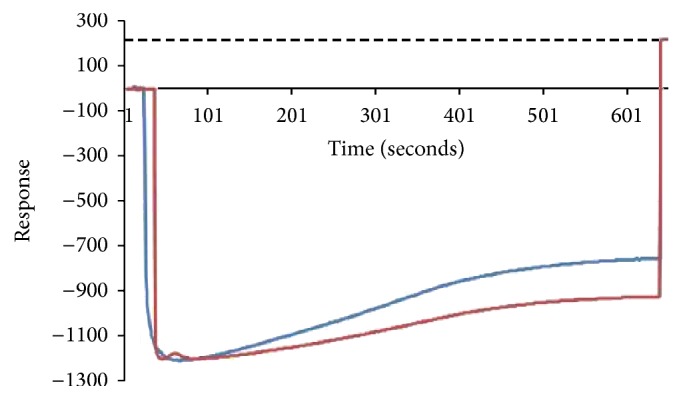
Sensorgram graph of H-2L^d^:Ig DimerX protein immobilization on a carboxylated surface sensor chip. Protein immobilization graphs are observed on channels 1 (dark blue) and 3 (dark red). The stabilized dissociation RU values were considered as baseline for posterior studies of DimerX interaction with synthetic peptides. The data are representative of two replicates.

The kinetic parameters of DimerX/peptides complexes were assessed, prior to the ex vivo assay with the mouse cells, to ensure that the assayed peptides could stably bind to the H-2 cleft of DimerX proteins. The DimerX proteins were immobilized onto the sensor chip using the EDC/NHS methodology for covalent binding and, thus, we assumed that the *α*1 and *α*2 domains of the recombinant H-2 were free to interact with the synthetic peptides in solution. The RU value obtained after DimerX immobilization was taken as reference to define the baseline to measure peptides binding (Figure 1).

With the data gathered from SPR analysis it was possible to evaluate the kinetics of the interaction between DimerX and the peptides. Moreover, it could be observed that the affinity complex formation was variable among the studied peptides (Table 1). Our results suggest that the tested peptides should be capable of stable binding to MHC H-2 L^d^ cleft and, therefore, of participating in antigen presentation processes. These hypotheses are further corroborated by the values of *k*_*d*_ observed for these peptides (varying between 0.047 s^−1^ and 0.123 s^−1^), which are similar to those described for actual T lymphocyte epitopes [21].

Additionally, the Δ*G*° values for the DimerX/peptides complexes were assessed, presenting values below zero, which is an indicative of the spontaneity of these complexes' formation (Table 1). However, it is important to consider that the actual cellular environment in which these complexes interact presents rather distinct physicochemical conditions compared to those observed in the SPR assays. In the cellular microenvironment, the conditions observed are of acidic pH, which is a condition that favors the binding and competition between different peptides to the MHC molecules [22–24], whereas, in the SPR assays, the molecules are tested in a neutral pH buffer. Nevertheless, the SPR results are relevant in a biological context, as the complexes formed in these assays present kinetic characteristics that should keep them stable on the surface of antigen presenting cells.

**Figure 2:**
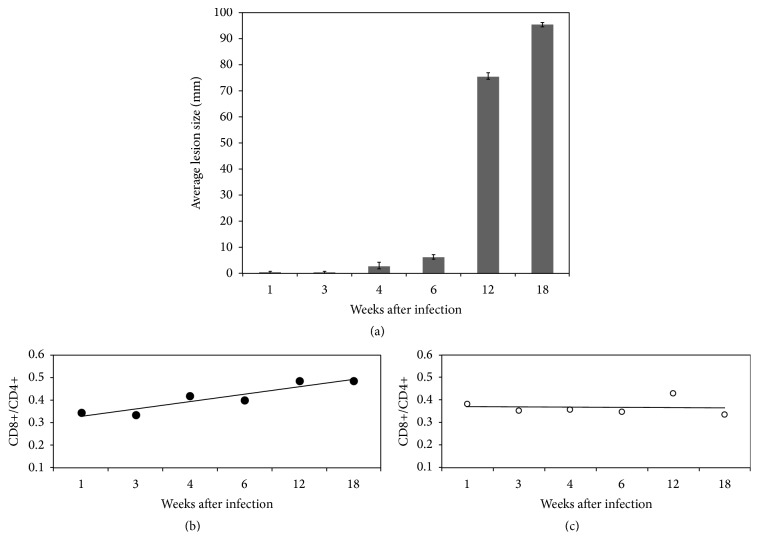
Profiles of lesion growth and CD4^+^/CD8^+^ T lymphocyte populations in BALB/c mice infected with* L. (L.) amazonensis*. Animals were inoculated subcutaneously with 1.0 × 10^5^ promastigotes in the plantar cushion of the left hind paw and lesions were monitored weekly by measuring the height (*h*) and width (*w*) of the cushion with a pachymeter (a). The results represent the lesion size (*h* × *w*) mean ± standard deviation (SD) of five animals, in millimeters (mm). The immunological profile of the popliteal lymph node cells was determined by flow cytometry in groups of infected (b) and noninfected (c) animals. The values of the CD8^+^/CD4^+^ ratio indicate an increase in the CD8+ lymphocyte population in animals infected with* L. (L) amazonensis*. The data are representative of three independents experiments performed in triplicate.

The paw lesions evolution and the CD4^+^ and CD8^+^ T lymphocyte population patterns in BALB/c mice infected with* L. (L.) amazonensis* were assessed and compared to a group of noninfected control animals. These data indicate that the infection (Figure 2(a)) modifies the lymphocyte population profiles causing an increase in CD8+ T lymphocyte population (Figure 2(b)), when compared to control mice (Figure 2(c)). The increase of CD8^+^ lymphocytes occurs early, in the first weeks of infection, when the lesion size is still discrete (Figure 2(a)) and remains increased throughout the whole analyzed time of infection (Figure 2(b)).

**Figure 3:**
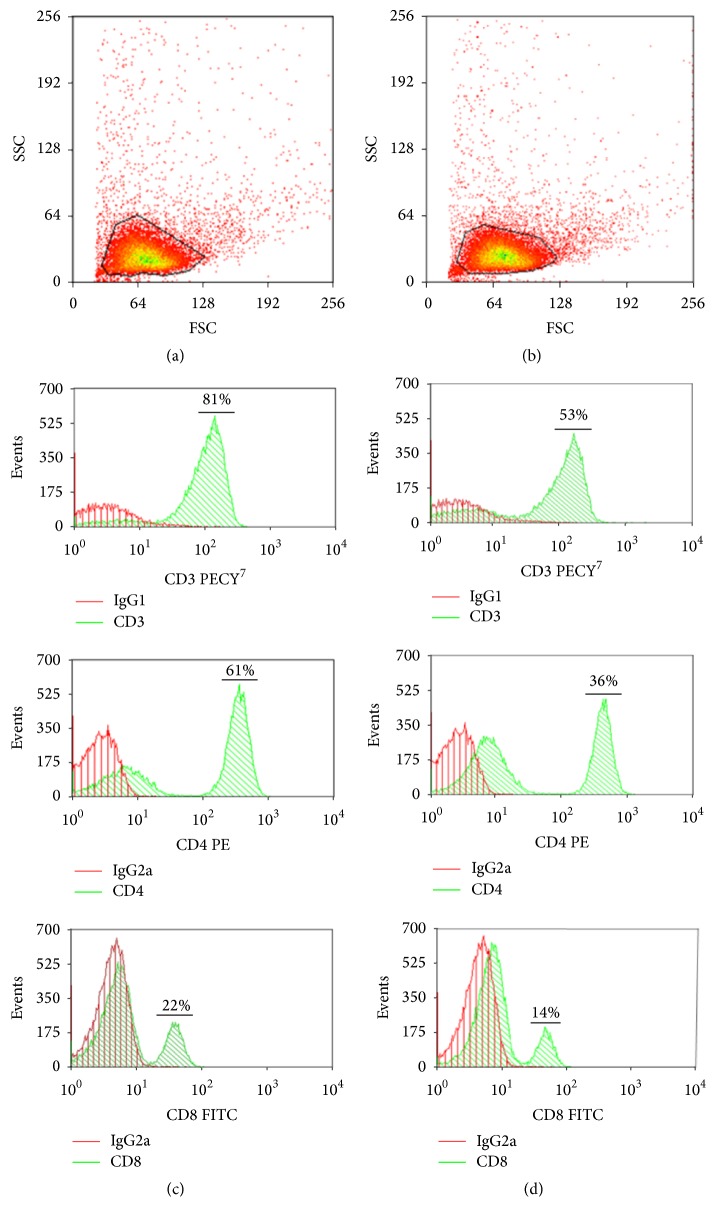
Graphic representations of flow cytometry. Dot plots were based on the evaluation of the gate analysis (a and b), delimiting the area of lymphocytes; we may infer that the morphology of the cells remained preserved throughout the study, which indicates that there were little cell death and suffering during the procedure to obtain them. The data are for the noninfected (a) and infected (b) mice group with* L. (L.) amazonensis*. Histogram superposition (c and d) shows the control isotype in the low fluorescence intensity, corresponding to negative region (0 to 10^1^), as signals above this range were considered positive reactions (10^2^–10^3^). This distance between areas of negative and positive signal is indicative of a good reaction receptor blocking unspecific binding. Moreover, the data show that there is a difference in the percentage of lymphocytes when comparing the noninfected (c) and infected (d) mice group with* L. (L.) amazonensis*. These data are representative of three replicates of lymph node cells analysis from 4th week of mice infection.

In addition, surface biosensing studies were performed to detect T lymphocytes reactive to the synthetic peptides, when coupled to the DimerX molecules. The lymphocytes samples used in these essays were previously evaluated which remained morphology preserved throughout the study, with little cell death and suffering during the handling (Figure 3). The used lymph node cells samples present differences in the percentage of CD4+ and CD8^+^ lymphocytes when compared to noninfected control mice samples (Figures 3(a) and 3(c)).

**Figure 4:**
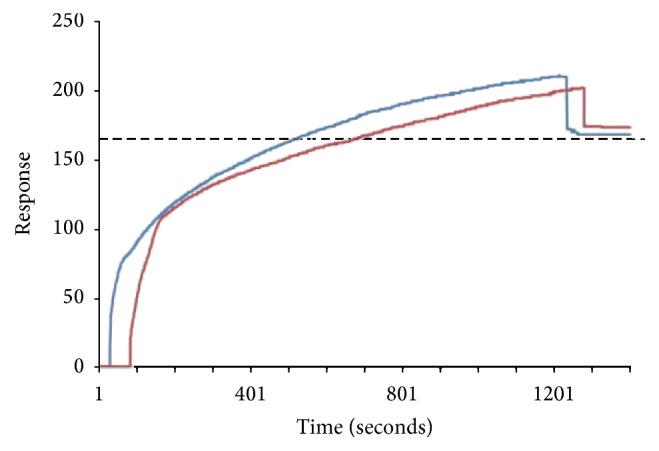
Sensorgram graph of DimerX interaction with BALB/c mice lymph node cells. The experiments were conducted with cells obtained from* L. (L.) amazonensis*-infected (dark blue) or noninfected (dark red) mice. The functionalized sensor chips were recovered with DimerX proteins without complexed peptides. The data are representative of two independents experiments performed in triplicate.

Lymph node cells were injected onto SPR chips covered by immobilized DimerX/peptides complexes (Figure 4). In these experiments, due to the complexity to define the kinetic parameters for whole cells, the RU values for association (RU_a_) and dissociation (RU_d_) were used as standards to assess interaction of cells with the complexes. Also, as the stability of RU_d_ signal over time relates to the stability of interaction, it was used as parameter to define the binding of reactive cells [17].

An assay was performed to assess the possibility of unspecific interaction of lymph node cells with the functionalized chip. In general, RU_a_ values ranged between 201 and 211 while RU_d_ values ranged between 168 and 173 in the experiments conducted with cells from infected or noninfected animals on chips containing only immobilized DimerX (Figure 4). These values are markedly distinct from those observed for tests with chips with immobilized DimerX/peptides complexes. In addition, it is relevant to notice that independent of their source (infected or noninfected mice) the cells presented no differences in their RU_a_ and RU_d_ values.

**Figure 5:**
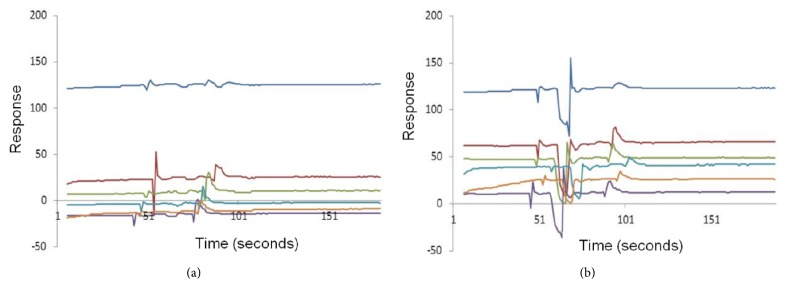
Interaction between immobilized DimerX/peptides complexes and lymph node cells preparations. The assays were performed with cells from* L. (L.) amazonensis-*infected (a) or noninfected (b) mice, which were streamed over a sensor chip containing the immobilized complexes. After each step of cell binding, the cells and peptides were removed with 30 mM HCl solution, leaving *α*1/*α*2 domains of the DimerX proteins free for a further interaction cycle. Sensorgram graphs are representative of all regeneration cycles (*n* = 120) and show the RU values for assayed peptides (P1.7 dark blue; P1.9 dark red; P1.10 green; P6.3 purple; P6.4 blue; P6.5 orange) during 151 seconds. The data are representative of three independents experiments performed in triplicate.

Throughout the study, after each test, the surface of the sensor chip was regenerated with HCl, as described in the methodology. Generally, the data responses indicated significant values (*p* < 0.05) of regeneration RU below the interaction H-2 L^d^:Ig/peptide for cells from noninfected and infected mice (Figure 5). From this data, it was possible to affirm that the *α*1/*α*2 domains of H-2 L^d^:Ig were set free for a new interaction.

Furthermore, experimental evidences support the hypothesis that the binding of the cells from infected mice to the immobilized complexes is specific, once the RU_d_ values obtained for these interactions were consistently higher that those observed for the interaction of cells from noninfected mice. The RU values obtained by the subtraction of the nonspecific interaction RU (cells from noninfected animals) from the specific interaction RU (cells from infected mice) were considered as representative of the binding effectiveness (Table 2). These effectiveness RU values allowed observing a different trend of T lymphocytes recognition for each peptide tested throughout the experimental infection timeline (Table 2).

Although T lymphocytes reactive to peptides P1.7, P6.3, and P1.10 have shown a clear tendency to diminish their reactivity along the infection, the cells reactive to peptides P1.9, P6.4, and P6.5 showed an oscillation in their reactivity, in general without a clear ascending or descending pattern. However, all reactive cells showed a reduction in their reactivity at the 18th week after infection. These data suggest that these peptides are of low immune significance in the late stages of infection, while they were much more significant, as determined by the number of reactive T lymphocytes, in the earlier infection stages. Their significance in this time point may render them as decisive factors for the establishment of the infection in BALB/c mice.

Moreover, it is also possible that the low detection of reactive T cells observed in the later stages of infection is due to the presence of lymphocytes primed to apoptosis, with an affected antigen recognition ability. Although the presence of such apoptotic T lymphocytes in mice lesions has already been reported [25], flow cytometry assays performed to confirm these cells in our lymph node preparation did not detect any such cells (data not shown).

The results presented herein correspond to a first surface biosensing-based approach applied to the detection of potential immunoregulatory T lymphocytes in experimental murine infection by* L. (L.) amazonensis*. Even though the SPR technique may present some limitations to detect whole cell due to their dimensions, which can lead to mass transport issues on the surface of the sensor chip, this methodology has been showing promising results in tracking cells with important biological activity, as recently described for detecting osteogenic cells [26].

The biosensing-based approach to determine the T lymphocyte phenotype proposed herein requires further discussion. The H-2 Ld:Ig construction was originally designed to study T lymphocyte function by immunofluorescence staining and flow cytometric analysis of antigen-specific T lymphocytes. In the present work, we devised an innovative application for this construction, applying it to the analysis of specific T lymphocytes, based on the concept that class I proteins bind to CD8+ T lymphocyte. Thus, the RU signal detected by using H-2 Ld:Ig/peptide complexes relates to CD8^+^ T lymphocytes, as the complex formed by class I protein and a binding peptide is recognized by and adheres to CD8^+^T lymphocyte [27].

It is also noteworthy that one of the advantages of the association of SPR with DimerX is the possibility of analyzing the binding phenomenon directly on the surface of lymphocytes, in real time. The data generated by this approach has the potential to reflect, in many aspects, the actual process of antigen recognition by T cells, as a fourth dimension (time) is considered in the establishment of molecular interactions [28]. Additional studies are needed to further refine these analyses into quantifying the lymphocytes clones present in the lymph node suspensions, as it has been proposed for the quantification of* Escherichia coli* [29].

## 4. Conclusions

We present a series of experiments to propose an innovative approach in immunoparasitology of* Leishmania*, using SPR-based methods for detecting antigen-reactive T lymphocytes in mice. The SPR data were useful to indicate that T lymphocytes specifically reactive to peptides from the region COOH-terminal cysteine proteinase B from* L. (L.) amazonensis* can be detected even in the early stages of the infection and present distinct variations in their populations throughout the infection. This fact is further evidence that cysteine proteinase B may contribute in interactions with the immune system of mice and affect the outcome of experimental infection.

## Figures and Tables

**Table 1:** Peptides from COOH-terminus region of *Leishmania (L.) amazonensis* CPB and data of H-2LD:Ig interactions.

ID	Sequences	MW	nmol	*k*_*a*_	*k*_*d*_	KD	Δ*G*
P6.3	EFCLGGGL	0794.25	0.61	2.20 × 10^8^	0.0560	00.25	−13.0
P6.4	CLGGGLCL	0734.20	0.54	1.20 × 10^7^	0.1089	09.00	−11.0
P6.5	EFCLGGGLC	0897.35	0.70	6.40 × 10^7^	0.0470	00.70	−12.5
P1.7	VMVEQVICF	1067.99	0.85	1.11 × 10^8^	0.1031	00.93	−12.3
P1.9	MVEQVICFD	0951.40	0.73	1.57 × 10^6^	0.1157	74.00	−09.7
P1.10	VEQVICFD	1082.35	0.84	1.10 × 10^8^	0.1230	01.00	−12.2

ID: identification of peptides; MW: molecular weight of peptides; nmol: molarity; association constant (*k*_*a*_; M^−1^ S^−1^); dissociation constant (*k*_*d*_; S^−1^); affinity constant (KD; nmolar); free Gibbs energy (Kcal/mol).

**Table 2:** Response data from subtractive dissociation between the response dissociation of cells from infected and noninfected mice with *Leishmania (L.) amazonensis*.

ID	RU_*s*_ 1st week	RU_*s*_ 3rd week	RU_*s*_ 4th week	RU_*s*_ 6th week	RU_*s*_ 12th week	RU_*s*_ 18th week
P1.7	161	102	96	115	DNS	0.0
P1.9	115	181	149	118	120	66
P1.10	DNS	227	DNS	123	31	44
P6.3	DNS	216	187	113	35	57
P6.4	DNS	100	127	140	DNS	66
P6.5	DNS	55	185	109	27	85

ID: identification of peptides; RU_*s*_: subtractive dissociation response; DNS: data not shown.
